# Cash transfers do not increase traumatic injury and mortality: evidence from Alaska

**DOI:** 10.1093/aje/kwag007

**Published:** 2026-01-29

**Authors:** Ruby Steedle, Robert E M Pickett, Tasce Bongiovanni, Hailie Dono, Byungkyu Lee, Erica Hobby, Anne Zink, Sarah K Cowan

**Affiliations:** Cash Transfer Lab, New York University, New York, NY, United States; Cash Transfer Lab, New York University, New York, NY, United States; Department of Surgery, University of California San Francisco School of Medicine, San Francisco, CA United States; Cash Transfer Lab, New York University, New York, NY, United States; Department of Sociology, New York University, New York, NY, United States; Cash Transfer Lab, New York University, New York, NY, United States; Department of Health, State of Alaska, Anchorage, United States; Cash Transfer Lab, New York University, New York, NY, United States; Department of Sociology, New York University, New York, NY, United States

**Keywords:** traumatic injury, cash transfers, Alaska

## Abstract

Direct cash transfers are an increasingly common tool to alleviate poverty, but critics argue that recipients will use this cash in irresponsible and potentially dangerous ways. To investigate the impact of cash payments on traumatic injury and death, we turn to Alaska’s long-standing statewide cash transfer, the Permanent Fund Dividend. Nearly every Alaskan receives a substantial amount of money (average $1500 per person) on a single day in the fall of each year. Using interrupted time series analyses paired with 2009-2019 data on all traumatic injuries (*n* = 36 556) seen at Alaska hospitals from the state’s trauma registry and all deaths (*n* = 43 170) from vital records, we examine whether the payment causes an increase in traumatic injury and mortality in the days following disbursement. Despite commonly held fears, we find no such increases in our data across multiple specifications. These results provide no evidence to suggest that direct cash payments increase the risk of injury or death.

## Introduction

Direct cash transfers are a long-standing, common and yet controversial tool used to combat poverty throughout the United States. Social Security, Temporary Aid to Needy Families, the Earned Income Tax Credit and Economic Impact Payments (stimulus payments) during the COVID-19 crisis are recent and notable examples. However, cash payments face strong scrutiny from critics who believe that recipients may spend the money on drugs and alcohol that could result in injury or death.^1-3^ Here, we ask whether this concern about harm from cash is well founded: Does a sudden influx of cash increase serious injuries or death?

We investigate whether cash payments increase traumatic injury and mortality in the short term by analyzing Alaska’s Permanent Fund Dividend (PFD). We examine this policy both because of its analytic properties and because of ongoing interest in its effects on injury and mortality in Alaska. Nearly every Alaska resident, including children, receives this annual dividend payment on a single day each fall. The payment size varies from year to year and averages $1500 per person during the study period. For many families, the PFD rivals support from cash and near-cash traditional safety net programs such as the Earned Income Tax Credit and the Supplemental Nutrition Assistance Program (food stamps). Unlike these means-tested programs, however, all Alaskans receive the same payment, regardless of income. Further, Alaskans receive the payment on the same day. These unique features allow us to measure the immediate effects of unearned cash transfers by comparing days before and after the cash disbursement.

We evaluate 2 hypotheses, as espoused by critics of cash transfer policies:

(H1) traumatic injury rates increase above expected levels in the days after the cash distribution; and

(H2) mortality rates increase above expected levels in the days after the cash distribution.

Permanent Fund Dividend payments could affect traumatic injuries and mortality through 2 potential mechanisms. First, the payment can generate a short-run uptick in general economic activity,^4^ such as more travel, work, shopping, and social outings, which could increase exposure to injury risk. Second, critics of unrestricted cash transfers often argue that some recipients may increase drug and alcohol consumption immediately after receipt, potentially elevating the risk of substance-use related injuries and deaths. In fact, substance-use related police reports increase in the days and weeks following PFD payments.^5^ This may be due to greater vigilance on the part of the police or increased substance use.

Prior evidence is mixed on whether these mechanisms translate into harms. Some studies found that an increase in economic activity, such as after monthly paydays, leads to increases in mortality^6^ and that an increase in substance-use-related hospital admissions follows the cash transfer of Supplemental Security Income in California^7^ and the 2008 Economic Stimulus Payments.^8^ Previous investigations into the relationship between the PFD and mortality found a correlation, however those findings were not statistically significant nor robust to multiple specifications.^9^ On the other hand, a randomized control trial of cash transfers in Chelsea, Massachusetts, showed the opposite: cash recipients were less likely to visit an emergency department overall or for substance use-related visits specifically.^10^ Here, we evaluate the downstream consequences of these 2 mechanisms on injury and mortality; examining the impact of cash on these mechanisms themselves, however, is beyond the scope of this manuscript.

## Data and methods

### Data

We analyze the short-term impact of PFD payments on severe health outcomes using 11 years of data on traumatic injury and mortality in Alaska. The Alaska Trauma Registry (ATR) compiles data on all traumatic injuries seen at Alaska emergency departments, defined as injuries that lead to hospitalization, observation, or death within 30 days of injury. Each observation in the registry is an emergency department visit, meaning individuals may appear in the data multiple times for separate hospital visits, and injuries that are not treated at a hospital and/or are minor enough to not require hospitalization are not included. The ATR contains 36 556 observations of Alaska residents injured within Alaska for 2009-2019.

We use Alaska Health Analytics & Vital Records Section records to examine the effects on mortality, which include a complete record of all 43 170 Alaska residents who died within Alaska from 2009 to 2019. Table 1 reports the demographic composition of the traumatic injury and mortality case data compared to the demographic composition of Alaska overall.

**Table 1:** Demographic composition of Alaska residents, Alaska Trauma Registry data, and Alaska mortality data.

	**Alaska**	**Traumatic injury data**	**Mortality data**
**Age**			
0-19	27.3	18.8	3.3
20-64	60.4	55.4	31.0
65+	12.2	25.8	57.4
**Race**			
White	63.7	55.2	68.0
American Indian/Alaska Native	15.5	36.6	24.4
Asian	6.1	2.3	3.5
Black	3.3	2.4	3.0
Hispanic	6.8	1.9	2.1
**Sex**			
Male	51.7	56.7	56.8

Combined with annual population data from the Alaska Department of Labor and Workforce Development, we constructed daily statewide trauma and mortality rates per 10 000 population. We do not use available data from 2020 or 2021 because of the unique context of the COVID-19 pandemic, including that Alaska distributed the PFD in early July 2020 (rather than October) making its effects indistinguishable from July 4th celebrations.

### Methods

We utilize Interrupted Time Series (ITS) models to compare observed trauma and mortality rates immediately following the cash distribution to the rates we would have expected in the absence of the payments. We simulate the expected rates based on the level and trends in those rates in the days leading up to PFD distribution.^11^ This method assumes that changes in trauma and mortality rates–compared to the rates we would have expected–occurring shortly following PFD distribution can be causally attributed to the cash transfer.

Our approach rests on 2 timing assumptions that we validate using both external research and sensitivity analyses. The first assumption is that the day of the PFD distribution is the treatment day. Nearly all Alaskans receive their PFDs on a single day but given the PFD is predictable and Alaskans have access to credit, they could in theory smooth their consumption in anticipation. This would, in effect, mean treatment occurs in advance of distribution for some Alaskans. Past research, however, has shown that Alaskans’ spending remains at normal levels in the days leading up to PFD distribution.^12^ We also test this assumption in a sensitivity analysis presented below which confirms use of the PFD distribution date as the treatment date.

The second timing-related assumption is that the increase in traumatic injury or mortality that interests us would occur shortly after the payment is made. Again, we turn to past research which shows Alaskans increase their spending for only 1 week following payments.^4^^,^^12^ As a robustness check we also examine a longer posttreatment window to ensure we are not failing to capture a delayed impact.

Having found support for both timing assumptions, we use pre-PFD distribution (ie, pretreatment) data to construct simulations to estimate the trauma and mortality rates we would have expected absent PFD payments. First, we use data from 30 days preceding PFD distribution to fit autoregressive negative binomial models. Because the unit of analysis is daily statewide traumatic injury and mortality rates, we do not include individual socio-demographic covariates but control for day of week effects, linear time trends, and year fixed effects (which accounts for the minimal changes in the demographic composition of the state over our analysis period) in our analyses. These models use lagged trauma and mortality rates in order to predict how a given day’s rate depends on that of the previous days (Equation 1). We used stepwise autoregressive model selection to identify the optimal number of autoregressive lags for each outcome, resulting in a 1-day lag in modeling mortality and 1- through 6-day lags in modeling traumatic injury. Model fit diagnostics, including Ljung-Box test statistics can be found in the Appendix (Tables S1 and S2).


(1) \begin{equation*} \log \left(E\left({Y}_t|X\right)\right)={\beta}_0+{\beta}_1t+{\sum}_{d=1}^D\beta{Y}_{t-d}+\omega +\alpha +\mathit{\log}(Population), \textrm{for}\ t<{t}_0\end{equation*}

where D = 6 for traumatic injury rates and D = 1 for mortality rates.

Using the models we fit on data up to the day before distribution, we then generate predictions of expected trauma and mortality rates for PFD distribution day and the next 7 days by simulating 1000 plausible rate trajectories. We do this by first simulating 1000 sets of coefficients based on the parameter means and variance–covariance matrix from the pretreatment models. We use these simulated coefficients and the true pre-PFD data to generate 1000 parameters ${\mu_t}^{\ast }$ for the predicted mean of the negative binomial distribution ${\mu_t}^{\ast }$.

Using the simulated ${\mu_t}^{\ast }$ parameters, we then draw random values from the negative binomial distribution with the dispersion parameter set to the estimate from Equation 1 to generate predicted values for daily trauma and mortality counts for days 0-7 post-PFD distribution. Using the predicted counts and the annual state population, we generate daily rates.

Finally, we compare the observed trauma and mortality rates on these days to each of these 1000 simulations and construct 95% Credible Intervals (CIs) for the effect of the PFD by calculating 2.5% and 97.5% quantiles of the difference between observed and simulated rates.

To validate that our approach has sufficient power to detect a theoretical change in traumatic injury and mortality rates, we conducted a sensitivity analysis using dates with known increases. Research outside of Alaska has shown that traumatic injury rates increase on New Year’s Day and the Fourth of July.^13^ Using each of these dates in place of PFD distribution day as *t_0_*, we confirmed that the 95% CIs generated by our models showed significant increases in both traumatic injury and mortality rates on these holidays (Appendix Figure S1).

We also tested a series of alternative model specifications and robustness checks.

*Alternative model specifications—presented in the Appendix:*

(1) Anticipatory shifted treatment dates: We also considered the possibility that anticipatory behavior could increase risk of injury or death in the days before payment distribution, thus biasing our simulations of expected outcomes in the absence of the PFD. To account for this, we tested “shifting” the treatment date in our models from the actual date of PFD distribution to a date 1 to 14 days pre-PFD distribution, and again found our results were consistent across anticipatory specifications (Figure S2).
(2) Pretreatment window choice: We examined the impact of choosing a 30-day pretreatment window by comparing the results of simulations fit on alternative windows, ranging from 15 to 80 days pre-PFD distribution, and found that the results were not sensitive to alternate window choice (Figure S3).
  (a) We additionally fit an extended pretreatment model using all available pre-PFD data, starting 30 days after the previous year’s PFD distribution. This model incorporated natural cubic splines to flexibly capture long-term temporal trends and Fourier series terms to model recurring seasonality and cyclicality; the results were consistent with our other models (Figure S4).

*Robustness checks—presented in the Results section below:*

(1) Unnatural death: We examine results for deaths by unnatural causes, which includes suicides, homicides, and accidents, separately. Either an increase in economic activity or an increase in drug- and alcohol-related spending would affect unnatural causes more than natural ones.

(2) Urban Alaska: Urban Alaska is quite similar to other small- and medium-sized cities across the United States. In order to enhance the generalizability of the findings, we narrow the analyses to urban Alaska, specifically Alaska’s most populous municipalities Anchorage and Fairbanks.

(3) Expanded analysis window after cash distribution: We expand our analysis window from 7 days post-PFD distribution to 30 days to test whether PFD payments result in delayed effects on traumatic injury and death.

## Results

**Figure 1:**
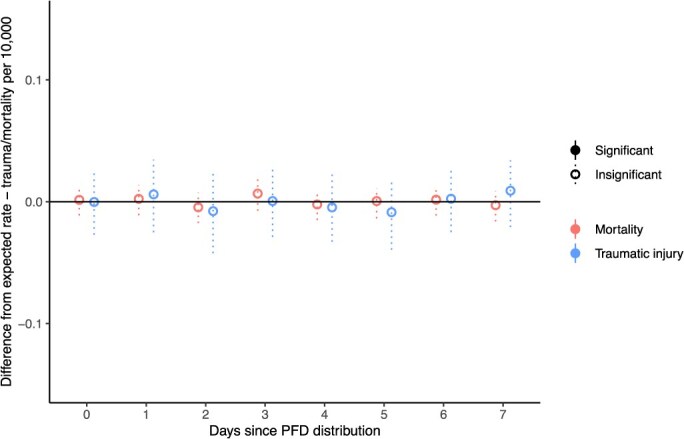
Difference between observed and simulated traumatic injury and all-cause mortality rates for 7 days post-cash distribution.

Our analysis shows that Alaskans were at no additional risk of traumatic injury or mortality on the day of payment distribution or for the following 7 days than they were before cash distribution, as can be seen in Figure 1. Figure 1 plots the mean differences between observed and expected traumatic injury and mortality rates per 10 000 population for PFD distribution day and the following 7 days as well as the 95% CIs. The effect estimate for traumatic injury on PFD distribution day is -0.0002 traumatic injuries per 10 000 population, which corresponds to 0.017 fewer traumatic injuries statewide, while the effect estimate for mortality on PFD distribution day is 0.0015 deaths per 10 000 population, corresponding to 0.110 additional deaths statewide. These effects are both very small and not statistically significant, as the 95% CI for both estimates covers 0. The traumatic injury and mortality effect estimates for days 1-7 post-PFD distribution are also all very small and not significantly different from 0.

**Figure 2:**
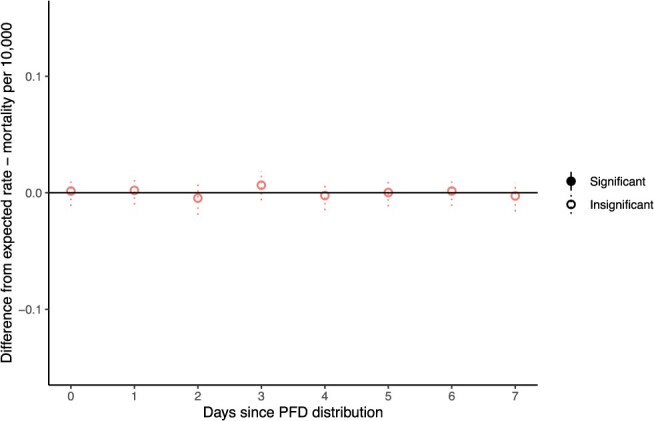
Difference between observed and simulated mortality rates for deaths by unnatural causes for 7 days post-cash distribution.

Several robustness checks confirm this result. First, we consider whether the null effect on all-cause mortality could be due to random variation in overall mortality rates concealing an effect on deaths from unnatural causes. To test this, we separately examine mortality from unnatural causes (Figure 2), defined as deaths due to suicide, homicide, or accidents, based on ICD-10 underlying cause of death code. Again, our analyses show no significant effect of PFD payments on mortality, regardless of cause of death.

**Figure 3:**
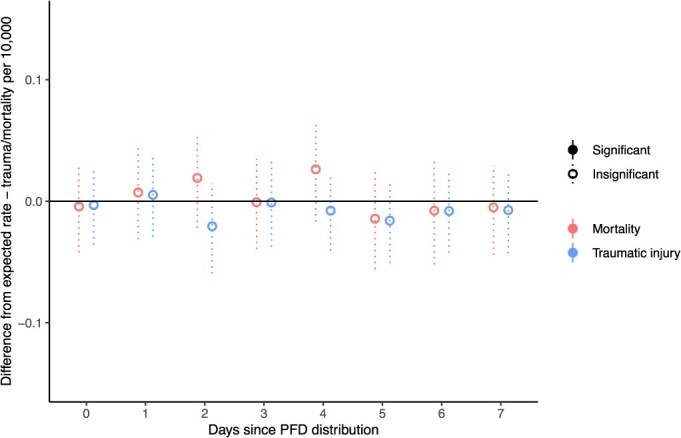
Difference between observed and simulated traumatic injury and mortality rates for metropolitan areas for 7 days post-cash distribution.

Next we explore whether the statewide null effect was consistent for urban Alaska. Restricting our sample to only injuries and deaths that occurred within the metropolitan areas of Anchorage and Fairbanks, our analyses again showed cash payments did not significantly increase rates of these events (Figure 3).

We also consider the possibility that our posttreatment analysis window is too short to detect an effect by expanding our analysis to 30 days post-PFD distribution to check for delayed spikes in traumatic injury or mortality rates.

**Figure 4:**
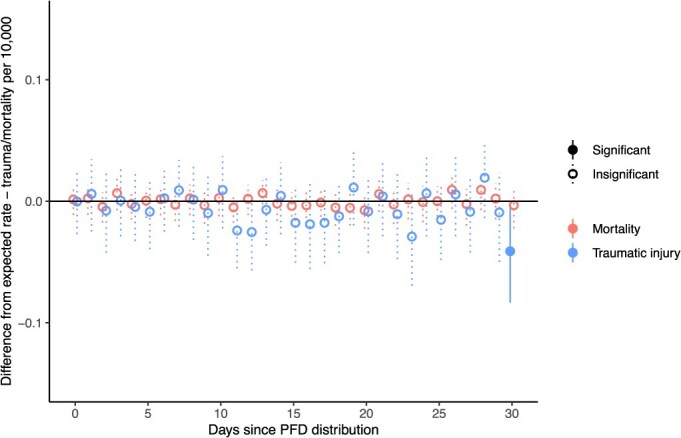
Difference between observed and simulated traumatic injury and all-cause mortality rates for 30 days post-cash distribution.

Figure 4 depicts 30-day results, which show no days where the traumatic injury or mortality rates were significantly higher than predicted ranges, supporting the findings of the 7 day window. The traumatic injury rate 30 days following PFD distribution is significantly lower than the predicted range, which again provides no evidence to support the hypotheses that traumatic injury or mortality rates increase following cash payments.

## Discussion

Cash transfers are an effective tool for reducing poverty, but their implementation is limited by critics who worry about irresponsible spending. Prominent policymakers, such as former Senator Joe Manchin, express concern that recipients spend their payments on drugs,^2^ and such behavior could lead to injury or death for themselves or others. Despite these fears, we find that Alaska’s annual cash distribution does not increase rates of serious traumatic injury or death in the short term. These results are consistent across numerous robustness checks; injuries and deaths do not increase in the 7 or 30 days following payments, or among urban residents, and deaths by unnatural causes specifically do not increase. Together, these findings provide strong evidence that narratives about short-term harm from cash payments are unfounded.

Our results also shed additional light on the findings of Evans & Moore^9^ who find suggestive evidence that the PFD increased mortality in some model specifications, whereas we find no evidence that the unearned cash increases traumatic injury or mortality in the days immediately following distribution. Our analysis differs from theirs in 2 ways: first, we have additional years of data. Second, given that there is no obvious counterfactual to Alaska, they use a variety of strategies to identify suitable comparisons and examine results across those comparisons. We instead turn to an internal control—Alaska before the payments. Consistent across these approaches is the result that there is little evidence of unearned cash being dangerous.

There are a few limitations of note in our analyses. Our design treats the entire state of Alaska as a single unit of analysis. While this approach allows us to measure the immediate effects of cash distribution by comparing days before and after disbursement, it also means our estimates could be affected by other statewide shocks or events that happen to coincide with PFD distribution timing. We also focus only on short-term impacts of the PFD in order to strengthen the causal validity of our identification strategy. Goods purchased with PFD payments could cause severe injury or death months after purchase, far beyond our analysis window. We are conceptually less concerned about attributing this to the dangers of the cash influx, however.

The trauma registry data we use comes from emergency departments, and includes only the most serious injuries that require hospitalization or lead to death. This excludes both minor injuries and the most severe cases where patients die before reaching the hospital. However, it is important to emphasize that our analyses of unnatural death, which capture severe injuries resulting in mortality irrespective of hospital admission, yield similar results, further supporting the robustness of our findings. While we are unable to measure the impact of cash on less severe injuries, the severe health outcomes that we focus on are of the greatest concern to policymakers.

We do not examine effects for any subgroups beyond urban Alaskans. We might expect larger shocks for lower-income Alaskans for whom the payment is a larger proportion of liquid assets, however adequate data for this analysis are not available at this time. Additionally, while we theorize substance use as a likely mechanism that could link cash payments to traumatic injury, high rates of missingness in substance use records prevent us from examining substance use as a contributing factor. We leave this for future research.

While we have addressed generalizability to other U.S. cities by our analysis of Alaska’s urban centers, the features of the PFD policy provoke other generalizability concerns. The universal and predictable nature of the PFD means that recipients may respond differently than they would to targeted or one-time transfers.

In sum, we find no evidence that cash payments increase risk of traumatic injuries or death. Across multiple robustness checks and alternative model specifications, rates of traumatic injury and death remained within predicted ranges in the week and month following cash distribution. This evidence that cash does not lead to severe harms complements the robust body of research documenting the numerous health benefits of long-standing cash programs such as the Earned Income Tax Credit and the PFD.^14-22^ Long-standing cash programs improve health throughout the lifecourse and as we show here, the evidence does not indicate they cause an increase in traumatic injury or mortality.

## Supplementary Material

Web_Material_kwag007

## Data Availability

We use restricted data provided by the Alaska Department of Health. The terms of our data sharing agreement do not allow us to make any of the data publicly available. Code to replicate these analyses is available at https://doi.org/10.58153/66xz8-zea47.
