# Supplementary material for: Cash transfers do not increase traumatic injury and mortality: evidence from Alaska
Source: Am J Epidemiol. 2026 Jan 29;195(5):1342–8. doi: 10.1093/aje/kwag007 (PMC13149014; doi:10.1093/aje/kwag007)
Supplement: Web_Material_kwag007 [file web_material_kwag007.zip › Trauma_AJE_appendix.docx]

**Cash Transfers Do Not Increase Traumatic Injury and Mortality: Evidence from Alaska**

**Supplementary Materials**

Ruby Steedle, Robert Pickett, Tasce Bongiovanni, Hailie Dono, ​​Byungkyu Lee, Erica Hobby, Anne Zink, and Sarah K. Cowan

**Included materials:**

- Appendix - Model validation and alternative specification robustness checks
  - Table S1 - Ljung-Box test statistics for negative binomial regression models of daily traumatic injury rates.
  - Table S2 - Ljung-Box test statistics for negative binomial regression models of daily all-cause mortality rates.
  - Figure S1 - Difference between observed and simulated traumatic injury and mortality rates for 7 days following New Years Day and Fourth of July holidays.
  - Figure S2 - Difference between observed and simulated traumatic injury and mortality rates for an anticipatory period and 7 days following PFD distribution, by choice of anticipatory period length.
  - Figure S3 - Difference between observed and simulated traumatic injury and mortality rates for 7 days following PFD distribution, by choice of pre-treatment analysis window length.
  - Figure S4 - Difference between observed and simulated traumatic injury and mortality rates for 7 days following PFD distribution, using longer pre-treatment analysis window and models including additional seasonality and cyclicality terms.

**Appendix - Model validation and alternative specification robustness checks**

***Model fit diagnostics***

We used Ljung-Box tests to determine the appropriate number of autoregressive lag terms to include in our negative binomial regression models for both traumatic injury and mortality data. Appendix Table S1 presents the Akaike Information Criterion (AIC), Correlated Akaike Information Criterion (AICc), Bayesian Information Criterion (BIC), and Ljung-Box test statistics for the traumatic injury data, and Appendix Table S2 presents the same for all-cause mortality data. Based on these tests, we included AR(1)-AR(6) lag terms in our traumatic injury models and only a single AR(1) lag in our mortality models.

***Table S1.*** *Ljung-Box test statistics for negative binomial regression models of daily traumatic injury rates.*

| **AR order** | **n** | **AIC** | **BIC** | **AICc** | **Deviance** | **DF residual** | **Ljung-Box test max lag** | **Ljung-Box p-value** |
| --- | --- | --- | --- | --- | --- | --- | --- | --- |
| **1** | 4016 | 21227.29 | 21277.67 | 21227.32 | 5207.61 | 4008 | 10 | 0.000 |
| **2** | 4015 | 21198.11 | 21254.79 | 21198.15 | 5180.24 | 4006 | 10 | 0.000 |
| **3** | 4014 | 21171.30 | 21234.27 | 21171.35 | 5155.37 | 4004 | 10 | 0.000 |
| **4** | 4013 | 21148.79 | 21218.06 | 21148.85 | 5134.12 | 4002 | 10 | 0.001 |
| **5** | 4012 | 21142.83 | 21218.40 | 21142.91 | 5130.11 | 4000 | 10 | 0.002 |
| **6** | 4011 | 21136.03 | 21217.89 | 21136.12 | 5125.65 | 3998 | 10 | 0.002 |
| **7** | 4010 | 21110.02 | 21198.18 | 21110.13 | 5102.12 | 3996 | 10 | 0.410 |
| **8** | 4009 | 21096.36 | 21190.81 | 21096.48 | 5088.47 | 3994 | 10 | 0.383 |
| **9** | 4008 | 21093.60 | 21194.34 | 21093.74 | 5087.36 | 3992 | 10 | 0.248 |
| **10** | 4007 | 21089.33 | 21196.36 | 21089.49 | 5084.57 | 3990 | 11 | 0.018 |

***Table S2.*** *Ljung-Box test statistics for negative binomial regression models of daily all-cause mortality rates.*

| **AR order** | **n** | **AIC** | **BIC** | **AICc** | **Deviance** | **DF residual** | **Ljung-Box test max lag** | **Ljung-Box p-value** |
| --- | --- | --- | --- | --- | --- | --- | --- | --- |
| **1** | 4016 | 12975.76 | 13026.14 | 12975.79 | 5086.91 | 4008 | 10 | 0.173 |
| **2** | 4015 | 12974.17 | 13030.85 | 12974.22 | 5085.94 | 4006 | 10 | 0.153 |
| **3** | 4014 | 12972.23 | 13035.21 | 12972.29 | 5084.00 | 4004 | 10 | 0.183 |
| **4** | 4013 | 12969.30 | 13038.57 | 12969.36 | 5079.06 | 4002 | 10 | 0.198 |
| **5** | 4012 | 12968.37 | 13043.94 | 12968.45 | 5076.14 | 4000 | 10 | 0.129 |
| **6** | 4011 | 12963.70 | 13045.56 | 12963.79 | 5071.47 | 3998 | 10 | 0.286 |
| **7** | 4010 | 12954.85 | 13043.00 | 12954.95 | 5064.10 | 3996 | 10 | 0.317 |
| **8** | 4009 | 12953.89 | 13048.34 | 12954.01 | 5061.14 | 3994 | 10 | 0.167 |
| **9** | 4008 | 12948.92 | 13049.65 | 12949.05 | 5054.16 | 3992 | 10 | 0.517 |
| **10** | 4007 | 12948.14 | 13055.16 | 12948.29 | 5054.00 | 3990 | 11 | 0.526 |

***Model validation - holiday analyses***

To validate that our models had sufficient power to detect a theoretical increase in traumatic injury and mortality rates, we conducted a sensitivity analysis using dates with known increases. Figure S1 depicts significant increases in both the traumatic injury and mortality rates on both New Years Day and the Fourth of July, mirroring the findings of Bergquist et al. (2020) [1].

***Figure S1.*** *Difference between observed and simulated traumatic injury and mortality rates for 7 days following New Years Day and Fourth of July holidays.*


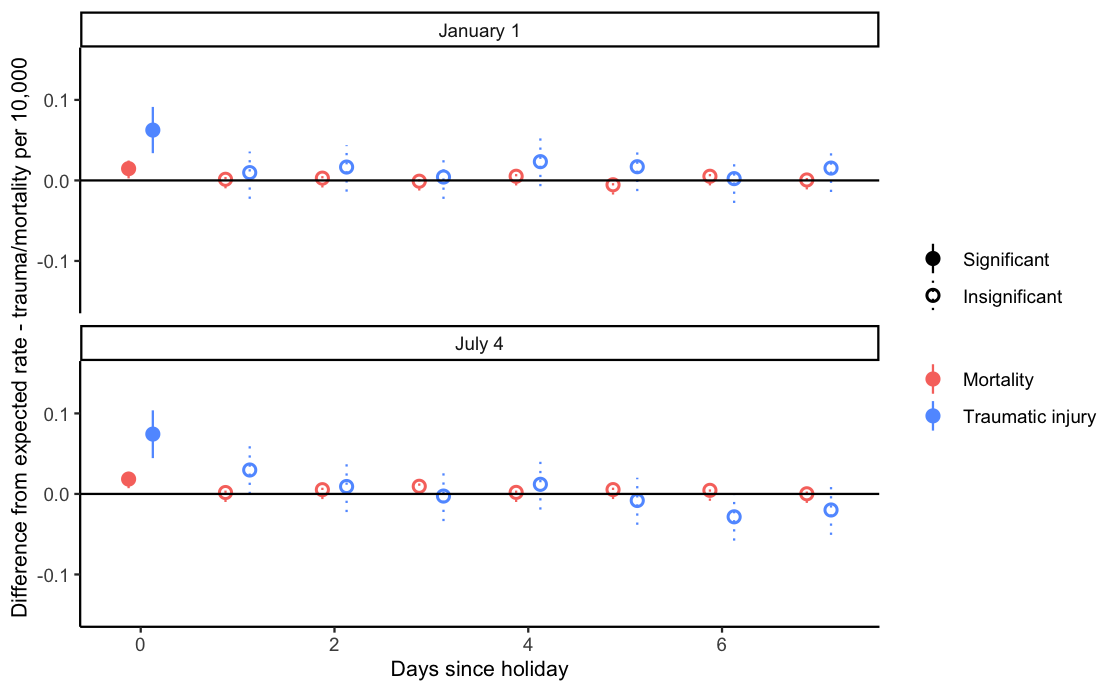


***Alternative model specifications - anticipatory shifted treatment dates***

We considered whether the lack of an increase in traumatic injuries and deaths after PFD distribution could be due to anticipatory behavior in the days leading up to PFD distribution, as both the date and amount of PFD payments are known in advance. Anticipatory behavior could increase the risk of injury or death in the days before payment distribution, biasing our simulations of expected outcomes in the absence of the PFD. To account for this, we tested “shifting” the treatment date in our models from the actual date of PFD distribution to one, seven, or 14 days pre-PFD distribution. We then fit models to only the pre-anticipatory period data, and simulated expected traumatic injury and mortality rates for both the anticipatory period and post-PFD distribution period. Figure S2 presents these analyses, which again show no significant increases in traumatic injury or mortality rates.

***Figure S2.*** *Difference between observed and simulated traumatic injury and mortality rates for an anticipatory period and 7 days following PFD distribution, by choice of anticipatory period length.*


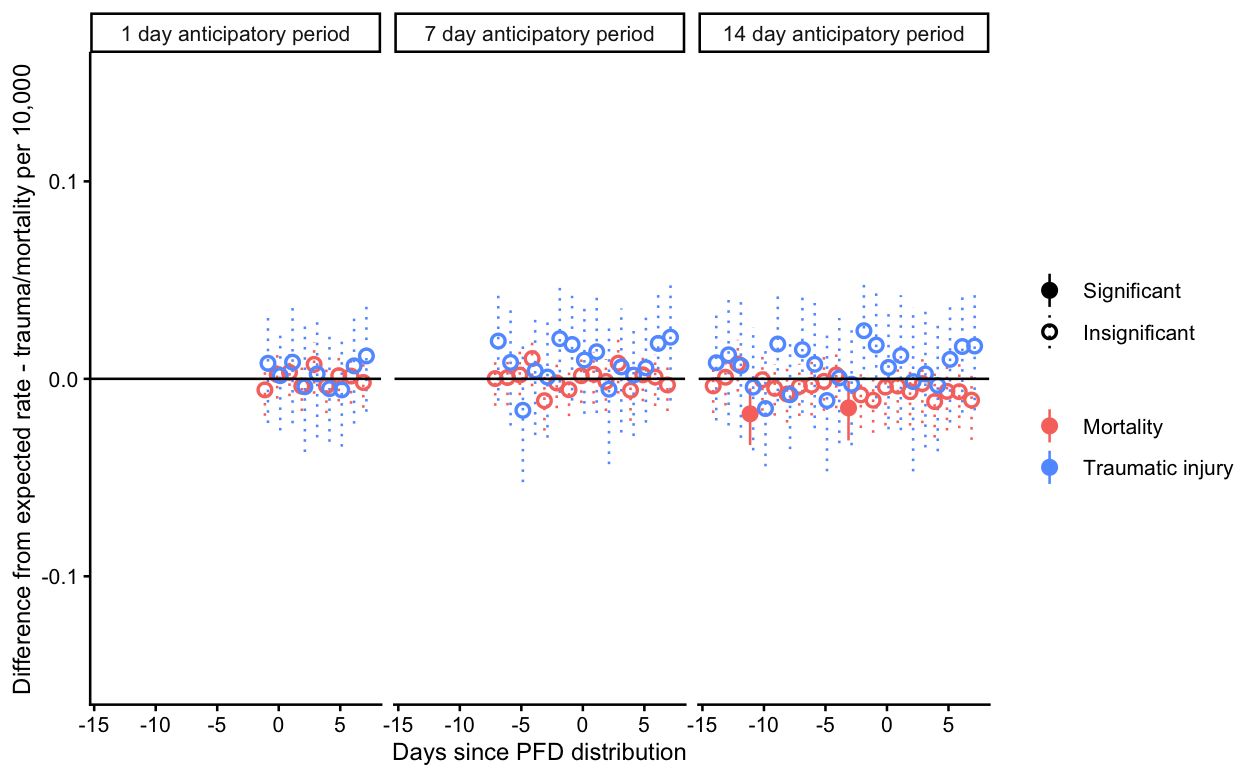


***Alternative model specifications - pre-treatment window choice***

We also evaluated whether the choice of a 30-day pre-treatment window substantively affects our findings. We tested this by fitting the same models to varying lengths of pre-treatment windows, ranging from 15 to 80 days pre-PFD distribution, and comparing the effect estimates produced by the resulting simulations. Figure S3 presents these comparisons, which show no significant deviations from expected traumatic injury or mortality rates regardless of pre-treatment window length.

***Figure S3.*** *Difference between observed and simulated traumatic injury and mortality rates for 7 days following PFD distribution, by choice of pre-treatment analysis window length.*

**
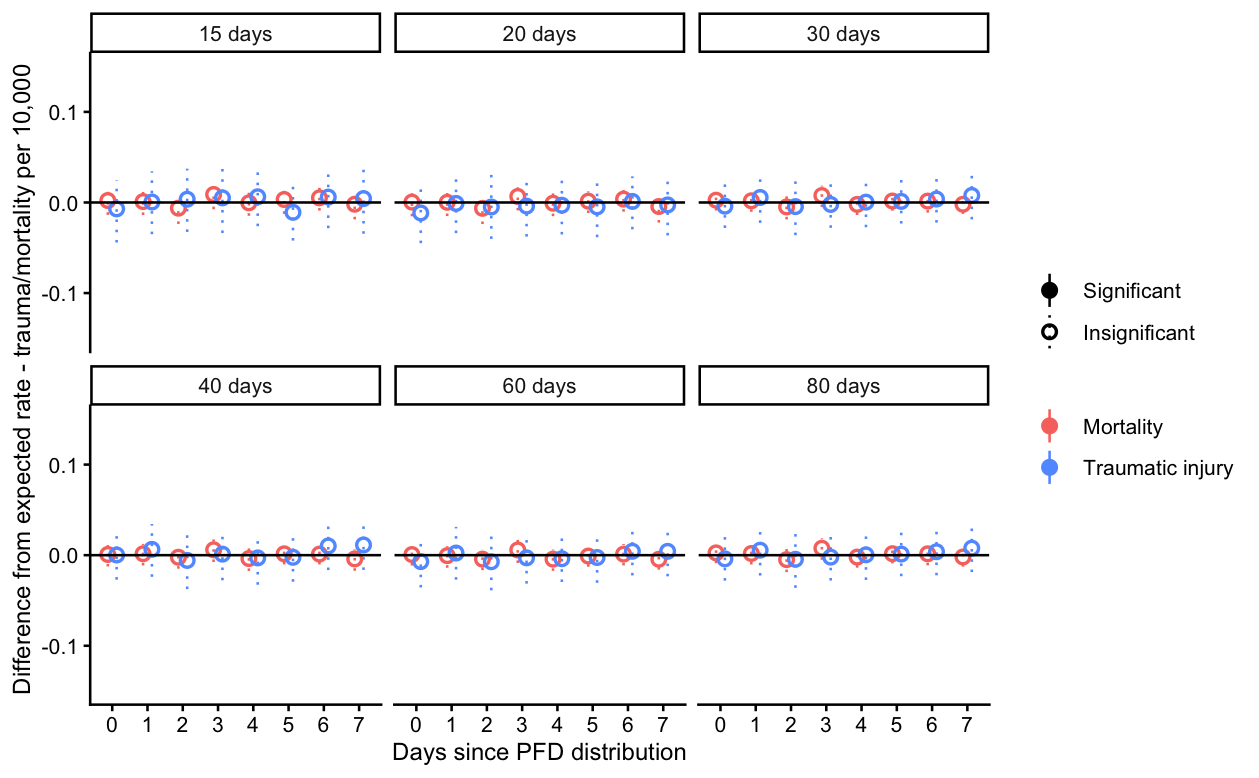
**

We further examined whether our models would benefit from fitting on an even longer period of pre-treatment data, with additional parameters for seasonality and cyclicality. To test this, we fit models using all days of pre-PFD data, starting 30 days after the previous year’s PFD distribution. To address the seasonality in traumatic injury and mortality rates, we included fifth-order natural splines and applied a Fourier transform in these models. We then followed the same simulation procedures to generate predicted rates for the week after PFD distribution and compared to observed rates. Figure S4 presents the comparison of observed rates to the rates predicted by these longer time series models, which again shows no significant deviations from expected rates.

***Figure S4.*** *Difference between observed and simulated traumatic injury and mortality rates for 7 days following PFD distribution, using longer pre-treatment analysis window and models including additional seasonality and cyclicality terms.*

***
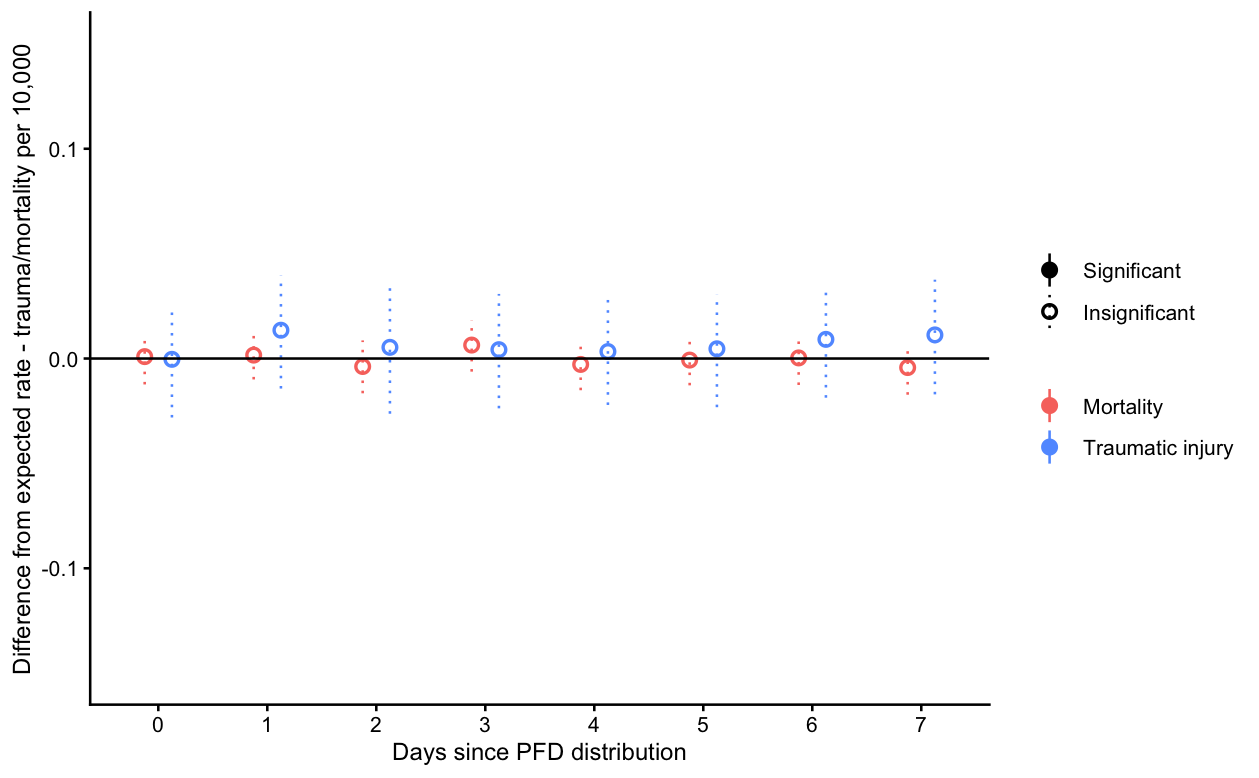
***

**References**

[1] Bergquist, T., Pejaver, V., Hammarlund, N., Mooney, S. D., & Mooney, S. J. (2020). Evaluation of the secondary use of electronic health records to detect seasonal, holiday-related, and rare events related to traumatic injury and poisoning. *BMC Public Health, 20(1)*, 46. https://doi.org/10.1186/s12889-020-8153-7
